# Medical Opinion and Movement

**Published:** 1906-08-25

**Authors:** 


### Medical Opinion and Movement.

Attention has been called recently to the practice adopted by one or two of the larger hospitals to advertise the institution by means of a statement at the meeting of governors setting forth the wonders of the treatment or the new methods of treatment practised in the institution. For more than a century it has been held to be contrary to the best traditions for the lay governors of a hospital in responsible charge of the management to advertise the practice of the medical staff attached to such an institution in this way. One result likely to arise is an increase in the amount of hospital abuse. We published in our columns a statement of the kind which was made by the chairman at a recent meeting, in which a great point was made of the opsonic method. "M.D." has written to the *Journal* to point out that a sister of a patient of his is receiving treatment at one of the special departments of one of our leading London hospitals. She or some member of the family had heard of the vaccine treatment combined with taking the opsonic index of the blood, and as she thought her condition was due "to her blood" she applied for the treatment and obtained it regularly and is still undergoing it in the special department of the hospital. The family are in affluent circumstances and could well afford to pay full medical fees. They live in a large well-kept house, with private grounds, and keep horses and carriages. Surely a direct instance of the cause and effect of medical abuse like the one we here call attention to should lead the chairmen of hospital meetings to be very careful to avoid advertisements of the doubtful character in question. The abuse of the hospitals is great enough at present in all conscience to render it desirable that everything possible shall be done by the responsible hospital authorities to diminish and not to increase its volume.

Dr. Donald F. Shearer insists that the ethical position of members of hospital staffs should be kept constantly in mind. The consultant or specialist who sees and continues to see and treat the patient of a general practitioner, without his knowledge, is looked at askance in private practice. He is considered to be acting in an undignified, if not actually dishonourable manner, but if he does the same thing in a hospital charity covers that sin as others. It is no doubt true that a patient has a right to choose his medical man, specialist or otherwise, but it can never be true that a patient has the right to expect secrecy if he accepts charitable relief, and the medical man who imposes or consents to such secrecy is outside the pale, whoever he may be. Many of the reputed consultants and specialists are so only in name, and are practically on a level with the general practitioner and take what comes. In their public offices the profession has a right to demand the strictest adherence to the recognised code of ethics. If the staff referred every hospital patient to his usual medical man at the first visit, abuses would cease and hospitals take their proper place among the most beneficent institutions of the world. It lies on the knees of the hospital staffs. We are inclined to agree with Dr. Shearer's view, but if it is to prevail the general practitioners must organise and assert themselves in a definite and practical manner. So long as they are content to permit their patients to be taken from them whenever the patient chooses to seek relief at a hospital, with the object of procuring treatment by a consultant, without paying a fee, general practitioners must expect that the difficulties they experience in getting a living in large cities especially must steadily increase until they may be robbed of their present means of livelihood altogether.

Dr. Langley Browne, Chairman of the Council, has, on behalf of the British Medical Association, addressed a letter to the clerk of the Privy Council asking that the period during which petitions may be presented to the Privy Council against the application for a charter on behalf of the Metropolitan Hospital Sunday Fund shall be extended. Dr. Browne, in his letter, represents that the British Medical Association as a body of 20,000 medical practitioners is deeply interested in a proposal which may have such important effects upon hospital management in the metropolis. He urges that owing to the holiday season and the absence of many members of the Association at the annual meeting at Toronto in Canada it is quite impossible to obtain any representative pronouncement of the opinion of the Association on the subject before September 3, the date fixed for giving notice of objection to the said petition. He therefore craves for further time for the presentation of objections in reference to this matter. The only ground advanced in support of this action on the part of the British Medical Association is that a first glance at the petition leaves an unfavourable impression, and it is urged not to be in the interests of the public that any steps should be taken to perpetuate the separate existence of the Hospital Sunday Fund at all. It is suggested that the Hospital Sunday, the Hospital Saturday, and King Edward's Hospital Funds should all amalgamate, and that they should collectively apply for a royal charter, when we gather the British Medical Association will be graciously pleased not to continue their opposition to the granting of such a charter. The next step rests with the Privy Council but we shall be surprised if that body considers the grounds advanced by Dr. Browne as outweighing the grave reasons which render it desirable that the decision of the Privy Council as to the granting of this charter to the Metropolitan Hospital Sunday Fund should be given before the end of October.

